# The Combined Impact of Female and Male Body Mass Index on Cumulative Pregnancy Outcomes After the First Ovarian Stimulation

**DOI:** 10.3389/fendo.2021.735783

**Published:** 2021-09-17

**Authors:** Zhonghua Zhao, Xue Jiang, Jing Li, Menghui Zhang, Jinhao Liu, Shanjun Dai, Hao Shi, Yuling Liang, Li Yang, Yihong Guo

**Affiliations:** ^1^Center of Reproductive Medicine, The First Affiliated Hospital of Zhengzhou University, Zhengzhou, China; ^2^Henan Key Laboratory of Reproduction and Genetics, Zhengzhou, China

**Keywords:** body mass index, assisted reproductive technology, cumulative live birth rate, male, overweight, obesity

## Abstract

**Objectives:**

To evaluate the combined impact of male and female BMI on cumulative pregnancy outcomes after the first ovarian stimulation.

**Design:**

Retrospective cohort study.

**Setting:**

University-affiliated reproductive medicine center.

**Patients:**

A total of 15,972 couples undergoing their first ovarian stimulations from June 2009 to June 2016 were included. During the follow-up period between June 2009 and June 2018, 14,182 couples underwent a complete ART cycle involving fresh embryo transfer and subsequent frozen embryo transfers (FETs) after their first ovarian stimulations. Patients with a BMI <24 kg/m^2^ served as the reference group. Patients with a BMI ≥ 24 kg/m^2^ were considered to be overweight, and those with a BMI ≥28 kg/m^2^ were considered to be obese.

**Intervention(s):**

None.

**Primary Outcome Measure:**

The primary outcome was the cumulative live birth rate (CLBR), which defined as the delivery of at least one live birth in the fresh or in the subsequent FET cycles after the first ovarian stimulation.

**Results:**

In the analyses of females and males separately, compared with the reference group, overweight and obese females had a reduced CLBR (aOR 0.83, 95% CI 0.7.92 and aOR 0.76, 95% CI 0.64–0.90). Similarly, overweight males had a reduced CLBR (aOR 0.91, 95% CI 0.83–0.99) compared with that of the reference group. In the analyses of couples, those in which the male was in the reference or overweight group and the female was overweight or obese had a significantly lower CLBR than those in which both the male and female had a BMI <24 kg/m^2^.

**Conclusions:**

The CLBR is negatively impacted by increased BMI in the female and overweight status in the male, both individually and together.

## Introduction

Overweight and obesity are defined as abnormal or excessive fat accumulation that threatens the health of the individual. A body mass index (BMI) over 25 kg/m^2^ is considered overweight, and a BMI over 30 kg/m^2^ is considered obese. The BMIs of Asian populations is generally lower than those of non-Asian populations (1). The World Health Organization (WHO) has predicted that approximately 20% of adults worldwide will be obese in 2025. An elevated BMI is a crucial risk factor for noncommunicable diseases, including cardiovascular diseases, musculoskeletal disorders and certain types of cancer. An increased BMI may also place women at risk for impaired fertility and adverse pregnancy outcomes, especially among couples seeking assisted reproductive technology (ART) (2–4). The obesity state can elevate proinflammatory adipokines through adipose tissue inflammation, such as interleukin-6 (IL-6), tumor necrosis factor-alpha (TNF-α) and free fatty acids (FFAs), which can induce both insulin resistance and compensatory hyperinsulinism (5). Hyperinsulinemia contributes to excess androgen, which is aromatized to estrogen in expansive adipose tissue (6). Raised estrogen levels lead to ovulatory dysfunction through a negative feedback mechanism within the HPO axis. The deleterious impact of a high female BMI on ART outcomes has been extensively studied (7) and systematically reviewed in the clinic (8). However, the couple rather than the individual is the object of interest in IVF treatment for sterile couples. Therefore, the importance of the male partner in couple fecundity should not be neglected, and assessing both male and female BMI is particularly necessary. Our previous study showed that couples with a higher female BMI had a lower live birth rate (LBR) than those with a normal BMI in IVF cycles (9). Similarly, Petersen et al. found that higher BMIs among couples negatively affect the LBR (10). Even if pregnancy is successful after ART treatment, McPherson et al. found that the combination of both maternal and paternal preconception overweight/obesity has a greater impact on infant birthweight (11). However, these studies were all limited by their lack of evaluations of the cumulative live birth rate (CLBR), an indicator of ART success that has been highly recommended in recent years (12). Furthermore, these studies have evaluated the relationship between BMI and CLBR in females only (13) while paying little attention to the relationship between BMI and CLBR in males (14). Therefore, we aimed to evaluate the combined impact of female and male BMI on cumulative pregnancy outcomes after the first ovarian stimulation.

## Materials and Methods

### Patients

This was a retrospective cohort study performed at a single reproductive medicine center of a university affiliated hospital fertility center. Data were collected from the Clinical Reproductive Medicine Management System/Electronic Medical Record Cohort Database (CCRM/EMRCD) at the Reproductive Medical Center, First Affiliated Hospital of Zhengzhou University, and the Henan Province Key Laboratory for Reproduction and Genetics. Cycles were excluded if either or both of the couples had an abnormal karyotype. We also excluded cycles with donor oocytes or sperm and excluded preimplantation genetic testing for aneuploidy (PGT-A) cycles, preimplantation genetic testing for monogenic/single gene defect (PGT-M) cycles, and preimplantation genetic testing for chromosomal structural rearrangement (PGT-SR) cycles. Cycles with no viable embryos were also excluded.

From June 2009 to June 2016, a total of 15,972 couples undergoing their first ovarian stimulation (IVF/ICSI) were screened for inclusion. Our follow-up period was from June 2009 to June 2018 with a minimum of 2 years of follow-up to observe whether the patients achieved live birth in the fresh cycle or subsequent frozen embryo transfer (FET) cycle. In total, 14,182 couples underwent a complete IVF treatment cycle during the follow-up period. A complete IVF treatment cycle was defined as achieving at least one live birth in the fresh or subsequent FET cycle with or without embryos remaining afterward or as not achieving a live birth after using all viable embryos. In total, 1,790 couples with remaining frozen embryos from the first ovarian stimulation discontinued fertility treatment due to personal factors after failing to achieve a live birth. Therefore, we analyzed cumulative pregnancy outcomes among the 14,182 couples. This study was authorized by the Institutional Review Board and Ethics Committee of the First Affiliated Hospital of Zhengzhou University. All the participants signed written informed consent forms.

### Dataset

BMI was calculated from information on weight and height at the initial consultation. According to the BMI guidelines for the Chinese population (1), we divided the female and male samples separately into three groups: reference group (BMI<24 kg/m^2^), overweight group (BMI≥ 24 kg/m^2^) and obese group (BMI≥ 28 kg/m^2^). For the couples analysis, we combined the females and males according to the BMI group.

### Ovarian Stimulation Schemes

The protocols were formulated according to the day of the patient’s menstrual cycle when she visited the hospital. A patient who was in the follicular phase was injected with triptorelin depot (decapeptyl 3.75 mg; Ipsen Pharma, France) intramuscularly on days 2–3 of the menstrual cycle. Pituitary downregulation was achieved after 28–42 days (E2 < 50 pg/ml, LH < 3 mIU/ml and ovarian cysts less than 10 mm). Patients who were in the luteal phase were injected with triptorelin (Ferring GmbH, 0.1 mg, Switzerland; Ipsen Pharma Biotech, 0.1 mg, France) intramuscularly during the midluteal phase, and 10 days later, the dose was decreased to 0.05 mg/d until pituitary downregulation (E2 < 50 pg/ml, LH<3 mIU/ml and ovarian cysts less than 10 mm) was achieved. Follicle-stimulating hormone (FSH) was used to start ovarian hyperstimulation (Gonal-F, Serono, Puregon, Netherlands, u-FSH, Livzon). The initial dose was dependent on the patient’s characteristics and antral follicle count (AFC), and the subsequent dose was adjusted according to follicle development and hormone levels. Human menopausal gonadotropin (HMG, Livzon) was added if needed. Oocyte maturation was triggered by 2000 IU of human chorionic gonadotropin (hCG, Livzon) and recombinant human chorionic gonadotropin (Merck Serono, Italy) when the maximal follicle diameter was more than 20 mm and when more than 2/3 follicles were >16 mm in diameter. Oocyte retrieval was performed 36–37 hours after hCG administration and with transvaginal ultrasound guidance. The insemination method was chosen based on sperm parameters. Then, the patient underwent embryo transfer (ET) (day-3 cleavage-stage embryos or day-5 blastocysts); however, patients at risk for ovarian hyperstimulation syndrome (OHSS), those with progestin suppression of the LH surge and those requiring fertility preservation underwent whole-embryo cryopreservation. Luteal phase support was sustained with progesterone vaginal gel (Merck Serono, Switzerland) at a dose of 90 mg/day from the day of ovum pick-up (OPU).

Endometrial preparation schemes for FET in the current study included natural cycles and artificial (estrogen (E)-P) cycles. The detailed procedures are described in our previous report (15).

### Outcomes

The primary outcome was the cumulative live birth rate, defined as the delivery of at least one live birth in the fresh or in the subsequent FET cycles, and only the first live birth event was considered in the analysis. Live birth was defined as the delivery of an infant after at least 24 weeks’ gestational age. The secondary outcome was the cumulative clinical pregnancy rate (CCPR) calculated based on observations of a gestation sac by B-mode ultrasound 35 days after ET.

### Statistical Analysis

SPSS (Statistical Package for Social Science, SPSS Inc., Chicago, IL, USA) version 26.0 was used for data analysis. Continuous variables are presented as the mean ± SD, and differences between groups were compared by means of one-way ANOVA. Categorical variables are presented as frequencies (percentages) and were compared using the chi-square test. All tests were two-sided, and statistical significance was defined as *P*<0.05.Pairwise comparisons between all adjacent groups were performed with the Bonferroni correction, and P<0.05/3 was set as a significant difference. Logistic regression was performed for the pregnancy outcomes. The results are presented as the adjusted odds ratio (aOR) and 95% confidence interval (CI).

## Results

**Figure 1:**
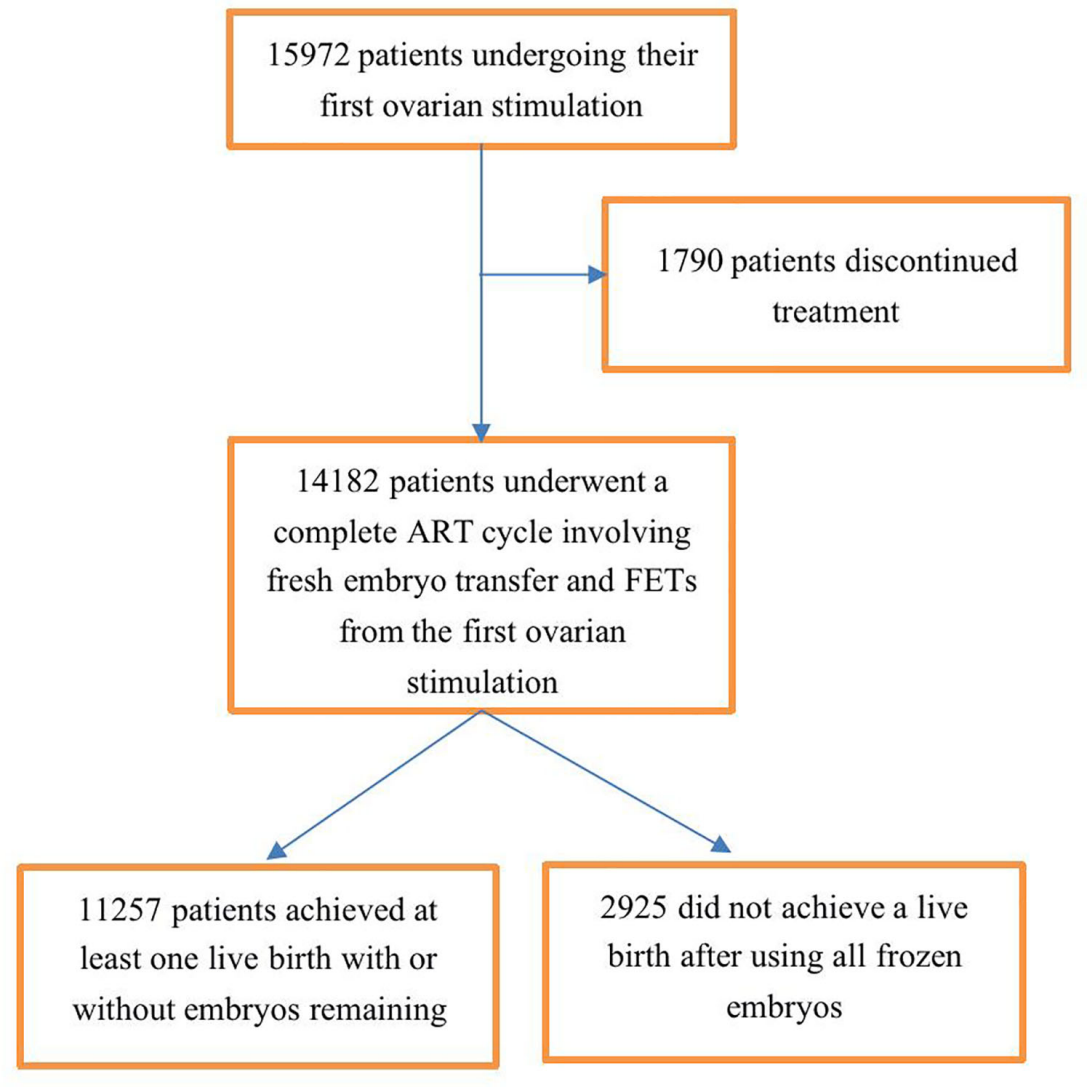
Flowchart of patient selection. A complete ART treatment cycle is defined as achieving at least one live birth in the fresh cycle or subsequent frozen embryo transfer (FET) cycles with or without embryos remaining afterward or is defined as not achieving a live birth after using all fresh and frozen embryos.

In total, 15,972 couples underwent their first ovarian stimulations, 14,182 (88.8%) of these couples underwent a complete ART cycle, and 1,790 (11.2%) discontinued fertility treatment. Therefore, we analyzed cumulative pregnancy outcomes among the 14,182 couples; of these couples, 11,257 achieved at least one live birth, and 2,925 did not achieve a live birth after using all of their frozen embryos from the first ovarian stimulation (**Figure 1**).

**Table 1** shows the clinical characteristics of the study population according to sex and BMI. The average age of the males was significantly higher in the overweight group than in the other groups (*P*<0.05). The average age of the females was significantly lower in the reference group (*P*<0.05), but there was no difference in age between the overweight group and obese group (*P*>0.05). Reference group showed higher baseline serum FSH and LH levels and lower AFC than the other groups(*P*<0.05). Regarding the cause of infertility, polycystic ovary syndrome (PCOS) was significantly more common among females in the overweight and obese groups than in the reference group (*P*<0.05).

**Table 1:** Clinical characteristics of the study population according to sex and body mass index.

		Reference	Overweight	Obese	*P* value
Female	N (total n=14,182)	10,268 (72.4)	3113 (22.0)	801 (5.6)	
	Age at start of 1st cycle (years)				
	Mean ± SD	30 ± 4.8	31.1 ± 5.1	31.0 ± 4.9	<0.001^a^ ^b^
	<30 years	5201 (50.7)	1266 (40.7)	327 (40.8)	<0.001^a^ ^b^
	≥30 years	5067 (49.3)	1847 (59.3)	474 (59.2)	
	Infertility cause, n (%)				<0.001^a^ ^b^ ^c^
	Tubal factor	4887 (47.6)	1387 (44.6)	336 (41.9)	
	Male	2483 (24.2)	667 (21.4)	167 (20.8)	
	PCOS	575 (5.6)	342 (11.0)	132 (16.5)	
	Uterine anomalies	277 (2.7)	112 (3.6)	26 (3.2)	
	Endometriosis	221 (2.2)	51 (1.6)	7 (0.9)	
	Ovarian disease	95 (0.9)	35 (1.1)	6 (0.7)	
	Missing observations	1730 (16.8)	519 (16.7)	127 (15.9)	
	Basal FSH (IU/L)	7.3 ± 2.5	6.9 ± 2.4	6.5 ± 2.0	<0.001^a^ ^b^ ^c^
	Basal LH (IU/L)	5.8 ± 3.5	5.4 ± 3.5	5.5 ± 4.1	<0.001^a^ ^b^
	Antral Follicle Count(AFC)	12.1 ± 0.1	12.7 ± 0.1	14.1 ± 0.2	<0.001^a^ ^b^ ^c^
Male	N (total n=14,182)	5845 (41.2)	5949 (41.9)	2388 (16.8)	
	Age at start of 1st cycle (years)				
	Mean ± SD	30.8 ± 5.4	32.4 ± 5.8	31.7 ± 5.3	<0.001^a^ ^b^ ^c^
	<30 years	2689 (46.0)	2023 (34.0)	891 (37.3)	<0.001^a^ ^b^ ^c^
	≥30 years	3156 (54.0)	3926 (66.0)	1497 (62.7)	
	Infertility cause, n (%)				<0.001^a^ ^b^
	Male	1510 (25.8)	1289 (21.7)	518 (21.7)	
	Female	3402 (58.2)	3617 (60.8)	1470 (61.6)	
	Missing observations	933 (16.0)	1043 (17.5)	400 (16.8)	

Data are presented as the mean ± SD or frequency (percentage).

The differences between groups (Bonferroni correction, P < 0.05/3) are indicated by the following superscripts:

a P: Comparison of reference and overweight groups.

b P: Comparison of reference and obese groups.

c P: Comparison of overweight and obese groups.

**Table 2** shows the treatment and pregnancy outcomes according to sex and BMI. Among females, the three groups were comparable in terms of the numbers of oocytes retrieved, insemination method and CCPR (*P*>0.05). The Gn dose differed between the groups, and the highest dose was administered in the obese group (*P*<0.05). The CLBRs of the overweight group and obese group were comparable (77.6% *vs.* 77.2%, *P*>0.05) and significantly lower than that in the reference group (80.1%, *P*<0.05). Among males, although the difference in terms of the CCPR and CLBR between the three groups was statistically significant per the chi-square test, there was no statistically significant difference between any two groups after the Bonferroni correction (reference *vs.* overweight *vs.* obese, CCPR: 84.0% *vs.* 82.8% *vs.* 84.8%, CLBR: 79.9% *vs.* 78.4% *vs.* 80.3%, *P<*0.05).

**Table 2:** Treatment and pregnancy outcomes according to sex and body mass index.

		Reference	Overweight	Obese	P
Female	Total gonadotropin dose (IU)	2032.9 ± 889.1	2276.0 ± 937.1	2507.8 ± 974.4	<0.001^a^ ^b^ ^c^
	No. of oocytes retrieved	12.4 ± 6.6	12.6 ± 6.9	12.8 ± 6.8	0.314
	Insemination method, n (%)				0.051
	IVF	7206 (70.2)	2257 (72.5)	590 (73.7)	
	ICSI	2897 (28.2)	811 (26.1)	200 (25.0)	
	IVF+ICSI	165 (1.6)	45 (1.4)	11 (1.4)	
	Cumulative clinical pregnancies per woman	8632 (84.1)	2579 (82.8)	653 (81.5)	0.066
	Cumulative live births per woman	8224 (80.1)	2415 (77.6)	618 (77.2)	0.003^a^ ^b^
Male	Insemination method, n (%)				<0.001^a^ ^b^
	IVF	4032 (69.0)	4322 (72.7)	1699 (71.1)	
	ICSI	1737 (29.7)	1525 (25.6)	646 (27.1)	
	IVF+ICSI	76 (1.3)	102 (1.7)	43 (1.8)	
	Cumulative clinical pregnancies per woman	4695 (84.0)	4924 (82.8)	2027 (84.8)	0.035
	Cumulative live births per woman	4467 (79.9)	4662 (78.4)	1919 (80.3)	0.039

Data are presented as the mean ± SD or frequency (percentage).

The differences between groups (Bonferroni correction, P < 0.05/3) are indicated by the following superscripts:

a P: Comparison of reference and overweight groups.

b P: Comparison of reference and obese groups.

c P: Comparison of overweight and obese groups.

**Table 3** shows the results of the multilevel analysis according to female and male BMI. After adjustments were made for confounders, an increased female BMI was associated with worse pregnancy outcomes after the first ovarian stimulation. The obese group had worse results than the overweight group. Compared with the reference group, the overweight and obesity groups had 17% (95% CI 0.75–0.92) and 24% (95% CI 0.64–0.90) reductions in CLBR and 12% (95% CI 0.79–0.98) and 25% (95% CI 0.62–0.90) reductions in CCPR, respectively. Similar tendencies were seen among males in the overweight group compared with males in the reference group, with a 9% (95% CI 0.83–0.99) reduction in CLBR. The effect of male obesity on the CLBR was not statistically significant (*P*>0.05).

**Table 3:** Results from logistic regression analyses of pregnancy outcomes in IVF/ICSI cycles according to sex and stratified by body mass index (BMI).

		CCPR		CLBR	
		Crude OR (95%CI)	Adjust OR (95%CI)	Crude OR (95% CI)	Adjust OR (95%CI)
		*P*-value	*P*-value	*P*-value	*P*-value
Female					
	Reference	1 (ref)	1 (ref)	1 (ref)	1 (ref)
	Overweight	0.92 (0.82-1.02) 0.106	0.88 (0.79-0.98) 0.021	0.86 (0.78-0.95) 0.002	0.83 (0.75-0.92) <0.001
	Obese	0.84 (0.69-1.01) 0.060	0.75 (0.62-0.90) 0.003	0.84 (0.71-1.00) 0.046	0.76 (0.64-0.90) 0.002
Male					
	Reference	1 (ref)	1 (ref)	1 (ref)	1 (ref)
	Overweight	0.91 (0.83-1.00) 0.061	0.92 (0.83-1.01) 0.075	0.91 (0.83-0.99) 0.029	0.91 (0.83-0.99) 0.037
	Obese	1.07 (0.93-1.22) 0.349	1.07 (0.94-1.22) 0.331	1.02 (0.91-1.15) 0.710	1.03 (0.91-1.16) 0.682

Female analyses adjusted for age, baseline serum FSH level,baseline serum LH level.

AFC and infertility cause.

Male analyses adjusted for age.

**Table 4** shows the results of the multilevel analysis of pregnancy outcomes based on the combined female and male BMI. The association persisted after adjustments for confounding factors. The reference group (couples with BMI<24 kg/m^2^) accounted for the largest proportion (31.3%). As shown in the findings from the separate analysis of females and males in **Table 3**, couples with an overweight or obese female (with any male BMI status) had a significantly lower CLBR than couples in which both the male and female had a BMI <24 kg/m^2^, but the reductions in aORs were not statistically significant for couples with male obesity. Among couples with a male BMI <24 kg/m^2^, those with female obesity had a significantly lower CLBR than couples with an overweight female [aOR (95% CI): 0.69 (0.52–0.92) *vs.* 0.83 (0.70–0.97), *P*<0.05]. Similarly, among couples with an overweight male, those with female obesity had a lower CLBR than those with an overweight female [aOR (95% CI): 0.73 (0.55–0.95) *vs.* 0.78 (0.67–0.90), *P*<0.05]. Couples consisting of an obese male and an overweight or obese female had a decreased CLBR compared with those consisting of a female from reference group [aOR (95% CI): 0.84 (0.68–1.04) *vs.* 0.84 (0.59–1.19) *vs.* 1.04 (0.90–1.21), respectively], though the *P* value was >0.05. The results were worse in the group in which both members of the couple were overweight, rather than only than female [aOR (95% CI): 0.78 (0.67–0.90) *vs.* 0.83 (0.70–0.97), *P*<0.05].

**Table 4:** Results from logistic regression analysis of joint couple BMI on pregnancy outcomes in IVF/ICSI cycles.

Combination of BMI (kg/m^2^)			CCPR				CLBR			
Female	Male	n (%)	n (%)	OR	95% CI	*P* value	n (%)	OR	95% CI	*P* value
Reference	Reference	4432 (31.3)	3745 (84.5)	1 (ref)	–		3577 (80.7)	1 (ref)	–	
Reference	Overweight	4251 (30.0)	3531 (83.1)	0.93	(0.82–1.04)	0.191	3360 (79.0)	0.93	(0.83–1.03)	0.160
Reference	Obese	1585 (11.2)	1356 (85.6)	1.10	(0.93–1.29)	0.264	1287 (81.2)	1.04	(0.90–1.21)	0.572
Overweight	Reference	1133 (8.0)	942 (83.1)	0.88	(0.73–1.05)	0.144	885 (78.1)	0.83	(0.70–0.97)	0.022
Overweight	Overweight	1383 (9.8)	1136 (82.1)	0.83	(0.71–0.98)	0.027	1061 (76.7)	0.78	(0.67–0.90)	0.001
Overweight	Obese	597 (4.2)	501 (83.9)	0.92	(0.72–1.16)	0.465	469 (78.6)	0.84	(0.68–1.04)	0.108
Obese	Reference	280 (2.0)	226 (80.7)	0.67	(0.49–0.92)	0.013	214 (76.4)	0.69	(0.52–0.92)	0.012
Obese	Overweight	315 (2.2)	257 (81.6)	0.75	(0.56–1.01)	0.060	241 (76.5)	0.73	(0.55–0.95)	0.022
Obese	Obese	206 (1.5)	170 (82.5)	0.80	(0.55–1.16)	0.231	163 (79.1)	0.84	(0.59–1.19)	0.325

Data are presented as ORs with 95% CIs.

Confounding factors included female age, male age, baseline serum FSH level, baseline serum LH level,AFC and infertility cause.

## Discussion

In summary, the major finding of this study was that both in the separate and combined analyses, increased female BMI and overweight in males adversely affected the cumulative pregnancy outcomes after the first ovarian stimulation, leading to decreases in the CLBR.

### Effects of Female Overweight/Obesity on the CLBR

In our study, females with an increased BMI had a significantly lower CCPR and CLBR, which showed a downward trend when the female was obese compared with when she was overweight. The negative effects of increased female BMI on pregnancy outcomes have been well established in prior work. For example, a 2019 systematic review showed that compared with normal-weight women, overweight women had a lower probability of giving birth following IVF [RR: 0.94; 95% CI: (0.91–0.97)], and women with obesity had a significantly lower LBR [RR: 0.85; 95% CI: (0.84–0.87)] (8). A similar result has been reported in other observational studies; specifically, Kawwass et al. (4) showed that in a retrospective cohort study (494,097 fresh autologous IVF cycles), compared with normal-weight women, women with obesity had a significantly lower probability of intrauterine pregnancy and live birth. In another retrospective analysis of 239,127 fresh IVF cycles, Provost et al. (7) reported that there was a significant decrease in CPR and LBR as BMI increased.

The aforementioned studies did not evaluate the association between female BMI and CLBR.A recent study about the correlation between female BMI and CLBR showed that the CLBR in overweight and obese patients decreased significantly compared with normal weight patients (13). In our previous study, we also found that overweight and obesity were associated with a decreased CCPR and CLBR in both women with PCOS and women with tubal factor infertility (16). Consistent with our findings, an American study conducted by Goldman et al. (17) showed that women with overweight, class III obesity or superobesity had progressively lower CLBRs [HR (CI): 0.96 (0.93–0.99), 0.76 (0.68–0.85), and 0.41 (0.26–0.63), respectively]. The same results were also reported by Toftager et al. (18) and Hu et al. (14) found that females with obesity had a lower CCPR and CLBR than females who were overweight.

### Effects of Male Overweight/Obesity on CLBR

Regarding males in this study, CLBR was significantly negatively influenced only by overweight. To our knowledge, the effect of male BMI on ART outcomes is contradictory, especially for CLBR. For example, some studies have reported the negative effect of a higher male BMI on pregnancy outcomes (9, 19), while Umul et al. (20) and Merhi et al. (21) have reported no effect. Hu et al. (14) found no significant correlation between paternal BMI and CLBR in a multiple regression model, whereas paternal overweight had a negative impact on the CLBR in women over 35 years old. Nevertheless, we failed to observe a significant difference in the effect of male obesity on cumulative pregnancy outcomes. It may be that the number of obese males was relatively small (41.2% in the reference group, 41.9% in the overweight group, and 16.8% in the obese group), which led to a statistically undetectable difference. Next, because of the inherent limitations of retrospective data, we were unable to set an exclusion criterion that was strictly standard for males, which may contribute to selection bias. In addition, we did not have information about sperm, which is a potential confounder affecting the CLBR (22).

### Effects of Combined BMI on CLBR

To date, studies of the synergistic effects of male and female BMI on CLBR are scarce. We extended the field and found that an increased female BMI had a negative impact on the CLBR regardless of whether the male was in the reference or overweight group. Additionally, the results worsen when both members of the couple are overweight rather than when only the female is. Ramlau-Hansen et al. (23) found that couples have a high risk of infertility if they are both obese. Setti et al. (24) observed that couples with a normal BMI had a significantly higher fertilization rate, high-quality embryo rate on day 2, blastocyst development rate, and implantation rate than couples in which at least one partner had an abnormal BMI (>24.9 kg/m^2^) in ICSI cycles. Similarly, an animal study based on diet-induced obese mice also showed that combined parental obesity led to a lower blastocyst rate and slower embryo development speed than single parental obesity (25). Regarding time-to-pregnancy (TTP) in couples, Sundaram et al. observed that couples whose BMIs were within obese class II (≥35 kg/m^2^) had a longer TTP than couples whose BMIs were <25 kg/m^2^ (26). Consistent with our study, a retrospective study showed that couples with a higher female BMI had a lower LBR than couples with normal weight after the IVF cycle, and no association was found in ICSI cycles (9), which was similar to the findings of Petersen et al. (10). They found that higher BMIs among members of the couple negatively affect the LBR. On the basis of the aforementioned studies, we further observed a cumulative negative effect of female BMI on the CLBR, namely, that female obesity had more negative effects than female overweight among couples with a male BMI in the reference or overweight group. The same cumulative negative effect of male BMI was achieved among couples with female overweight and obesity.

### Strengths and Limitations

The major strengths of our study are its ability to fill a gap in the existing literature by examining the joint effect of male and female BMI on cumulative pregnancy outcomes after the first ovarian stimulation. Second, our study had a large sample size, which allows for more exact estimates of outcomes. Nevertheless, there are some limitations in our study. First, this was a retrospective design that included a single medical center. Second, the data for smoking status, alcohol intake, metabolic health of the patients and whether diabetes was present was not recorded and their influence cannot be eliminated, which weakens the generalizability of the findings. Additionally, the lack of detailed data of male also weakens the universality of conclusions. Therefore, we urge caution in interpreting the study results.

## Conclusion

In conclusion, the results of our study indicate that an increased BMI in females and overweight in males, both independently and combined, negatively impact the cumulative pregnancy outcomes after the first ovarian stimulation, leading to a lower CLBR. Therefore, effective management of the couple’s BMI, such as weight loss and lifestyle changes, might help to improve pregnancy outcomes. With the joint action of the members of the couple, it will be easier to implement these changes and more effectively reach an ideal BMI.

## Data Availability Statement

The original contributions presented in the study are included in the article/supplementary material. Further inquiries can be directed to the corresponding author.

## Ethics Statement

The studies involving human participants were reviewed and approved by the Institutional Review Board and Ethics Committee of the First Affiliated Hospital of Zhengzhou University. The patients/participants provided their written informed consent to participate in this study.

## Author Contributions

ZZ, XJ, and YG contributed to the conception and design of the study. JL, MZ, and JHL were responsible for the data collection and checking. ZZ and XJ performed the data analysis, interpretation and manuscript drafting. SD and HS assisted in the data analysis. YL, LY, and YG supervised the project administration and assisted in writing the paper. All authors contributed to the article and approved the submitted version.

## Funding

Supported by the National Natural Science Foundation of China (Grant No. 81571409) and the Program for Innovative Scientific Research Team of Henan Province of China (Grant No. 18IRTSTHN030).

## Conflict of Interest

The authors declare that the research was conducted in the absence of any commercial or financial relationships that could be construed as a potential conflict of interest.

## Publisher’s Note

All claims expressed in this article are solely those of the authors and do not necessarily represent those of their affiliated organizations, or those of the publisher, the editors and the reviewers. Any product that may be evaluated in this article, or claim that may be made by its manufacturer, is not guaranteed or endorsed by the publisher.
